# European *Chlamydia abortus* livestock isolate genomes reveal unusual stability and limited diversity, reflected in geographical signatures

**DOI:** 10.1186/s12864-017-3657-y

**Published:** 2017-05-04

**Authors:** H. M. B. Seth-Smith, Leonor Sánchez Busó, M. Livingstone, M. Sait, S. R. Harris, K. D. Aitchison, Evangelia Vretou, V. I. Siarkou, K. Laroucau, K. Sachse, D. Longbottom, N. R. Thomson

**Affiliations:** 1 Pathogen Genomics, Wellcome Trust Sanger Institute, Wellcome Trust Genome Campus, Hinxton, Cambridgeshire CB10 1SA UK; 2 Moredun Research Institute, Pentlands Science Park, Bush Loan, Edinburgh, Midlothian EH26 0PZ UK; 3 Retired: Formerly Laboratory of Biotechnology, Department of Microbiology, Hellenic Pasteur Institute, Athens, 115 21 Greece; 4 Laboratory of Microbiology and Infectious Diseases, School of Veterinary Medicine, Faculty of Health Sciences, Aristotle University of Thessaloniki, GR-54124 Thessaloniki, Greece; 5 Anses, Animal Health Laboratory, Bacterial Zoonoses Unit, University Paris-Est, 14 rue Pierre et Marie Curie, 94701 Maisons-Alfort, France; 6 Friedrich-Loeffler-Institute (Federal Research Institute for Animal Health), Institute of Molecular Pathogenesis, Naumburger Str. 96a, 07743 Jena, Germany; 7 Current Address: Universitätsspital Basel, 4031 Basel, Switzerland; 8 Current Address: Applied Microbiology Research, Department of Biomedicine, University of Basel, 4031 Basel, Switzerland; 9 Current Address: Microbiological Diagnostic Unit, Department of Microbiology and Immunology, Peter Doherty Institute for Infection and Immunity, The University of Melbourne, Parkville, VIC 3010 Australia; 10 Current Address: RNA Bioinformatics and High-Throughput Analysis, Faculty of Mathematics and Computer Science, Friedrich-Schiller-Universität, Leutragraben 1, 07743 Jena, Germany

**Keywords:** Single nucleotide polymorphisms, Multi Locus Variable Number Tandem Repeat, Multi Locus Sequence Typing

## Abstract

**Background:**

*Chlamydia abortus* (formerly *Chlamydophila abortus*) is an economically important livestock pathogen, causing ovine enzootic abortion (OEA), and can also cause zoonotic infections in humans affecting pregnancy outcome. Large-scale genomic studies on other chlamydial species are giving insights into the biology of these organisms but have not yet been performed on *C. abortus*. Our aim was to investigate a broad collection of European isolates of *C. abortus*, using next generation sequencing methods, looking at diversity, geographic distribution and genome dynamics.

**Results:**

Whole genome sequencing was performed on our collection of 57 *C. abortus* isolates originating primarily from the UK, Germany, France and Greece, but also from Tunisia, Namibia and the USA. Phylogenetic analysis of a total of 64 genomes shows a deep structural division within the *C. abortus* species with a major clade displaying limited diversity, in addition to a branch carrying two more distantly related Greek isolates, LLG and POS. Within the major clade, seven further phylogenetic groups can be identified, demonstrating geographical associations. The number of variable nucleotide positions across the sampled isolates is significantly lower than those published for *C. trachomatis* and *C. psittaci*. No recombination was identified within *C. abortus*, and no plasmid was found. Analysis of pseudogenes showed lineage specific loss of some functions, notably with several Pmp and TMH/Inc proteins predicted to be inactivated in many of the isolates studied.

**Conclusions:**

The diversity within *C. abortus* appears to be much lower compared to other species within the genus. There are strong geographical signatures within the phylogeny, indicating clonal expansion within areas of limited livestock transport. No recombination has been identified within this species, showing that different species of *Chlamydia* may demonstrate different evolutionary dynamics, and that the genome of *C. abortus* is highly stable.

**Electronic supplementary material:**

The online version of this article (doi:10.1186/s12864-017-3657-y) contains supplementary material, which is available to authorized users.

## Background

The genus *Chlamydia* comprises an expanding group of obligate intracellular Gram-negative bacteria that are responsible for a broad range of disease in humans, other mammals and birds [1, 2]. Genomic sequencing of the genus [3–10] shows that they all share common features, including small, syntenic genomes, which are largely free of mobile elements. Several large scale studies have described the phylogenetic relationships of strains of the human pathogen *Chlamydia trachomatis* and the avian and zoonotic pathogen *Chlamydia psittaci* [11–13]. These analyses have begun to show the extent of diversity within each species and have revealed unexpectedly high levels of recombination and its importance for the ongoing evolution of these obligate intracellular organisms.

*Chlamydia abortus* (formerly *Chlamydophila abortus*) is an economically important pathogen of livestock, causing abortion and the premature birth of stillborn or weak offspring [1, 14], with economic implications for the farming industry. While the pathogen is endemic in small ruminants worldwide, it can also cause infection in cattle, pigs, deer and horses, amongst other host species. The organism is zoonotic and can cause spontaneous abortion in pregnant women, as well as being potentially fatal for the pregnant mother [15]. Disease caused by *C. abortus* is variously referred to as ovine enzootic abortion (OEA), enzootic abortion of ewes (EAE) or ovine chlamydiosis. Protection can be administered through commercial live-attenuated vaccines (Enzovax, MSD Animal Health; Cevac Chlamydia, Ceva Animal Health) or inactivated vaccines (e.g. Mydiavac, Benchmark Animal Health), although the live vaccine has been implicated in disease in a number of vaccinated animals [16, 17].

To date seven *C. abortus* genome sequences have been published [6, 9, 18]. The UK strain S26/3 was the first reference genome, comprised of a 1.1 Mb chromosome and, unlike other *Chlamydia*, no virulence-associated plasmid [18]. Two Greek isolates, LLG and POS, originating from the aborted fetuses of a goat and sheep respectively, represent the most diverse variant strains identified to date, with a further closely related strain recently described [19]. Two molecular typing schemes exist, using either multiple locus variable number tandem repeat analysis (MLVA), which has only identified seven MLVA sequence types (MTs) [19, 20], or multiple locus sequence typing (MLST) [19, 21], where only six MLST sequence types (STs) have been defined. This is in sharp contrast to greater diversity in other species of *Chlamydia* [21–23]. Studies on limited numbers of samples suggest that *C. abortus* isolates in livestock appear to be largely monomorphic: low diversity is observed throughout the genome, even within the plasticity zone (PZ), a region of high genomic variation in other chlamydial species [6, 9, 18].

The aims of this study were to understand the population structure and diversity of *C. abortus* circulating in livestock at the highest resolution, and to better understand their phylogeographic distribution. In order to address these issues, we present an analysis of the largest sample of sequenced genomes within a chlamydial species to date, comprising 64 isolates of *C. abortus*, predominantly isolated from European farm animals and African ruminants.

## Methods

### Cell culture, DNA extraction and sequencing

The isolates and sources of *C. abortus* used in this work are summarized in Additional file 1. Cell culture and DNA extraction was performed as previously described [9] and genomes were sequenced using the Illumina Genome Analyzer II or Hiseq 2000 with paired end 75, 100 or 108 bp reads. Read data for all samples sequenced has been submitted to the European Nucleotide Archive (ENA, http://www.ebi.ac.uk/ena/) under accession numbers given in Additional file 1.

### Mapping, assembly, annotation and plasmid detection

The genome of *C. abortus* S26/3 (GenBank accession CR848038) was used as a reference for the mapping of all *C. abortus* sample reads using SMALT [https://sourceforge.net/projects/smalt/] and a minimum identity threshold for mapping of 80%, resulting in the coverage statistics detailed in Additional file 1. GATK was used for indel realignment and single nucleotide polymorphisms (SNPs) were called using SAM/BCFtools. No mixed infections were identified, following analysis of heterogeneous variant sites. The avian *C. psittaci* strain 6BC (accession CP002586) was used to define the root of the phylogeny. Assembly of all genomes was performed using a high throughput assembly method [24], producing contigs of both the *C. abortus* genome and remnant cell line contamination (submitted with read data as above). Annotation from S26/3 was transferred to the genomes of AB7 and 1B using AnnotateBacteria [https://github.com/sanger-pathogens/Bio-AutomatedAnnotation] and manually curated using Artemis and ACT [25, 26]. The published genome and annotation of LLG [6] was updated with the current Illumina data, which provided greater coverage (140×).

All annotated genomes (S26/3, LLG, AB7, 1B) [Seth-Smith, H.M.B. et al: Genomic evidence that the live Chlamydia abortus vaccine strain 1B is not attenuated and has the potential to cause disease, in preparation] were curated for the presence of pseudogenes. Pseudogenes were defined as having one or more mutations that would ablate expression (i.e. indel causing frameshift or substitution causing premature stop codon). Functional versions of the 51 identified pseudogenes were used to create a database against which all sequenced genomes with paired-end data were queried for the presence of the 51 identified pseudogenes using ARIBA v2.4 [https://github.com/sanger-pathogens/ariba], with manual curation of some lower coverage genomes, especially with respect to phase-variable genes.

To identify any reads mapping to the chlamydial plasmid, the plasmids of *Chlamydia caviae* strain GPIC (accession AE015926) and *Chlamydia felis* strain Fe/C-56 (accession AP006862) were used as references against which to map all *C. abortus* sequence data, as well as read data from *C. psittaci* strain 01 DC12 [27] as a positive control. *C. caviae* and *C. felis* should be equal phylogenetic distances from both *C. psittaci* and *C. abortus* [28]. While mapping was seen from the *C. psittaci* sequence data (of 89.1% coverage at 423× and 89.6% coverage at 406× against *C. caviae* and *C. felis* respectively), none of the *C. abortus* isolate sequencing data produced coverage of either plasmid.

### SNP detection, phylogenetic reconstruction and clustering

A SNP alignment was obtained from the multiple genome alignment generated from mapping. A phylogenetic reconstruction of the data was carried out using RAxML [29] using a Generalized Time Reversible (GTR) model of evolution with a γ correction for among-site rate variation with four rate categories and 100 bootstraps. Strains were clustered into genetically close groups using hierBAPS [30] by performing two clustering iterations.

### Recombination and temporal analysis

To investigate the effect of recombination on the phylogeny, ClonalFrame v1.2 [31] and Gubbins [32] [https://github.com/sanger-pathogens/gubbins] were used. Results from Gubbins were explored using Phandango [https://github.com/jameshadfield/phandango]. The correlation between root-to-tip distances and date of isolation was used to explore temporal signal in the data for the whole collection and for the two BAPS clusters within the major clade independently. This analysis was performed using the “clustered permutation” approach implemented in R scripts [33]. Three instances of BEAST v1.8.2 [34] were run in parallel for the cluster with significant temporal signal (PG 2, see Results) using a GTRGAMMA model of evolution, strict molecular clock and constant population size for 100,000,000 generations.

### Analysis of population structure and diversity

The existence of population structure regarding host, geographical location or date of isolation was explored using the Discriminant Analysis of Principal Components (DAPC) implemented in the *adegenet* R package [35]. A randomization of the populations (host, country or date) was performed to assess statistical significance and clustering power. The diversity between and within geographical locations was further assessed using an Analysis of Molecular Variance (AMOVA), implemented in the *poppr* R package [36]. A permutation test was used to assess statistical significance (*n* = 1000). Nucleotide diversity was also calculated for the strains isolated in the different countries, as implemented in the *pegas* R package [37].

## Results

### The phylogeny of *C. abortus*

In order to gain a snapshot of the genomic diversity and phylogeny of *C. abortus* in livestock, we sequenced the complete genomes of a panel of 57 new isolates from farmed animals across Western and Mediterranean Europe, ruminants from Africa and two strains from the USA. In addition to the existing sequences [6, 9, 18], including the reference genomes S26/3 and LLG, this resulted in a set of 64 strains for analysis (Additional file 1). The majority of isolates were from intensively farmed animals presenting with clinical disease.

**Fig. 1:**
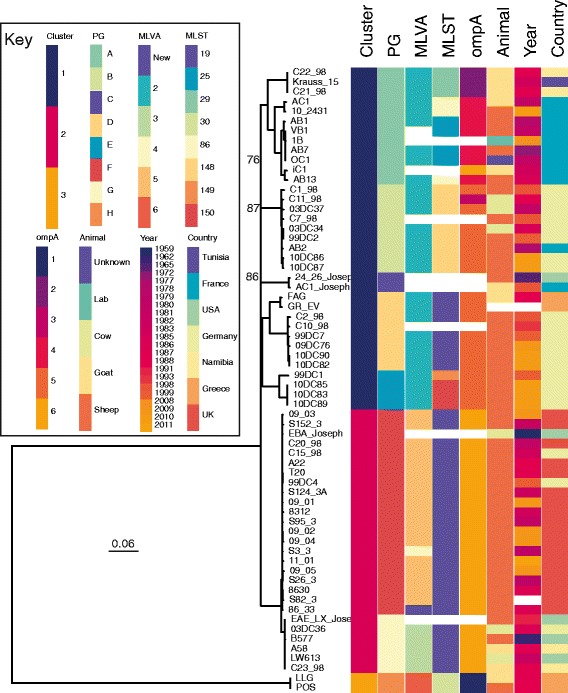
Phylogeny of *C. abortus*. Phylogenetic tree of all sequenced isolates included in this study, showing that the variant LLG and POS strains are deeply separated from all other isolates. *C. psittaci* was used to locate the position of the root of the tree. Three population clusters can be identified, with the major clade (clusters 1 and 2) further divided into 7 PGs. MLVA and MLST data is provided for comparison with the phylogeny, where it could be extracted from the data, otherwise is shown as a white track. Bootstraps on the major nodes under 100% are shown; bootstraps within PGs are often lower. Scale bar shows number of substitutions per site. Figure was drawn using Phandango

Visual inspection of the whole genome SNP-based phylogeny of *C. abortus* isolates (Fig. 1) shows that there are two deeply branching lineages of *C. abortus*. The smaller of these comprises the two previously defined variant strains LLG and POS [38, 39], separated from the larger lineage (denoted the ‘major lineage’) by approximately 3,800 SNPs (compared to the reference strain S26/3). The genomes of LLG and POS appear to be identical to each other, within the regions mapped to the reference genome used to detect variation (99.2%). Moreover, a direct comparison of these two genomes shows no SNPs between them across assembled regions, which cover the whole genome with the exception of regions of *CAB1*_*0289*-*90* (*pmp12*-*14*) and *CAB1*_*0613*-*4* (*pmp16*-*17*). Within our sequenced collection, no intermediate strains occur between LLG/POS and the major lineage. The major lineage includes all other sequenced isolates included in this study, with the range of SNPs between any one isolate and the reference S26/3 being between ten and 663 SNPs (the most distant strain being C2_98), and the range of SNPs separating pairs of isolates within the major lineage being from zero to 724 SNPs.

To unambiguously subdivide the species we used the software package Hierarchical Bayesian Analysis of Population Structure (hierBAPS) to identify robust Phylogenetic Groups (PGs) within the *C. abortus* phylogeny. hierBAPS identified three primary clusters, subdividing the major lineage into two distinct clusters, 1 and 2, which are further subdivided into seven subclusters or PGs (Fig. 1). It is clear from Fig. 1 that the branches representing five of the PGs within the major lineage (A, B, C, D/E and F/G) split at the same time from the same ancestral node, exhibiting polytomy.

To investigate whether there are associations between these clusters and those identified through MLVA and MLST typing schemes, respectively MTs and STs were predicted in silico from the assembled sequence data and compared against the defined PGs (Fig. 1). While both typing schemes agree broadly with the phylogeny, there are some notable exceptions, including a lack of resolution for MT2, covering the diversity represented by PGs A-E; and ST19, covering PGs D, F and G. Additionally, there are several examples of multiple MTs/STs within a single PG, with MTs 4, 5 and a new MT all represented within PG F; STs 25, 29 and 86 within PG A; 149 and 150 both within PG E.

### Recombination and diversity within *C. abortus*

**Fig. 2:**
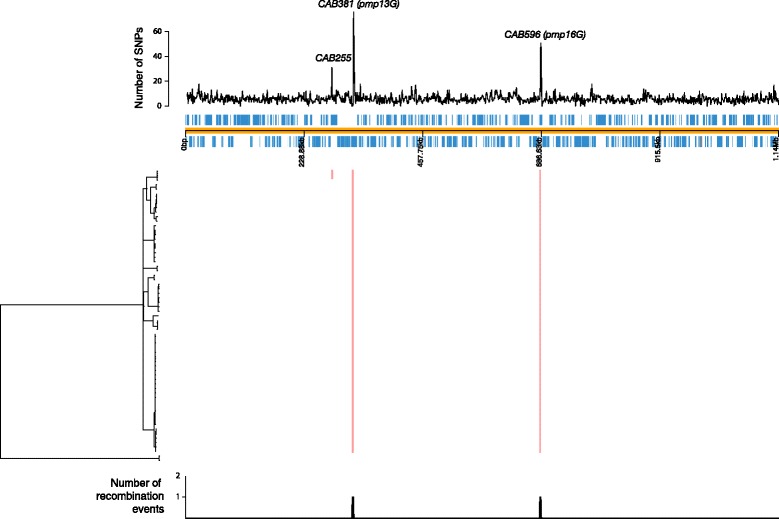
Plot of phylogeny (left) against recombinations predicted by Gubbins and SNP density. The reference genome of S26/3 is shown as an *orange* line with predicted CDSs in the forward and reverse frames shown in *light blue* above and below this line. Above this is the SNP density plotted with a window size of 1 kb. Peaks at *CAB255* (putative membrane protein), *CAB381* (*pmp13G*) and *CAB596* (*pmp16G*), the sites of recombination, can be seen. These recombination events were the only ones identified by Gubbins. Figure was drawn using Phandango

Recently it has been shown that recombination is an important feature of chlamydial evolution [11–13]. Recombination was analysed within our *C. abortus* dataset using Gubbins and ClonalFrame which both utilize SNP density to identify recombination blocks. Both algorithms produced very similar outputs with only three small regions of recombination identified (Fig. 2). These regions are located within *CAB255*, encoding a putative membrane protein (affecting PG C), and the polymorphic membrane protein-encoding genes *pmp13G*-*pmp14G* (*CAB281*-*282*) and *pmp16G* (*CAB596*) (affecting the major clade). This indicates that these latter recombinations are ancestral events that occurred after the divergence of the major clade and cluster 3 (LLG/POS), but before the expansion of the current major clade. There is no other evidence of subsequent recombination among isolates within the major clade. However, since SNP density is used to predict recombination, and the SNP density across the genome of *C. abortus* is relatively low for a chlamydial species, it is not possible to rule out the occurrence of undetected recombinations. Importantly, no mutations affecting recombination-associated *rec* genes, potentially impacting on the ability of *C. abortus* to recombine, were identified (Additional file 2).

Homoplasic SNPs are also markers of recombination [40]. A detailed assessment of the presence of homoplasic SNPs showed that only 0.9% of SNPs (59/6718 sites) are homoplasic, in contrast to 26% in *C. trachomatis*, which is known to be recombinogenic [11]. Investigation of these SNP loci showed that 25 of these sites fall within four polymorphic membrane protein genes, *pmp3E*, *pmp11G*, *pmp13G* and *pmp16G*, of which 14 are non-synonymous; eight are intergenic, and 16 further SNPs are predicted to result in amino acid changes in CDSs other than the *pmp*s (Additional file 3). The SNPs located in *pmp* genes could possibly result from mis-mapping or intragenomic recombination, and may not therefore represent true recombination within this species.

Due to this apparent lack of recombination, this dataset provides an opportunity to investigate SNP distribution in chlamydial isolates that has arisen only through mutation and selection. A very even SNP distribution is apparent across the genome (Fig. 2), with elevated SNP densities found at three locations which cover genes only around *CAB255*, *CAB381*, *CAB596* and *CAB673*, encoding a putative membrane protein, polymorphic membrane proteins Pmp13G and Pmp16G and a hypothetical protein, respectively. Surprisingly no elevated levels of SNP variation were seen across the PZ (*CAB539*-*543*) or the *tmh*/*inc* locus (*CAB760*-*775*).

The *ompA* gene (*CAB048*), often used for genotyping in *Chlamydia* species, shows a very limited variation among these *C. abortus* isolates. Only seven variable nucleotide sites were found to distinguish *ompA* in S26/3 from that in LLG, and only six allelic variants of *ompA* were evident within the whole tree. The *ompA*-genotypes do not reflect the population structure (Fig. 1), and this is therefore not a useful typing target in this species.

### Whole gene variation among *C. abortus* isolates

**Fig. 3:**
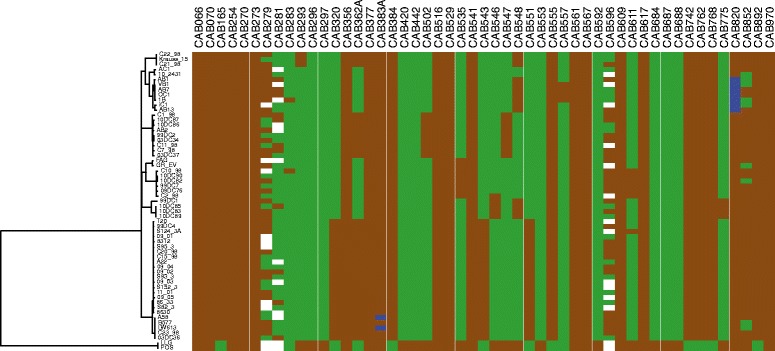
Pseudogene distribution in *C. abortus*. The distribution of each of the 51 identified pseudogenes within the phylogeny is shown, with functional genes shown in *green* and pseudogenes shown in *brown*. Genes which are predicted to have reverted from non-functional to wild-type sequence are shown in dark *blue*. Data with assembly uncertainty is shown in *white*, with genes containing longer homopolymeric tracts often showing variation within the reads. Figure was drawn using Phandango

Previous whole genome analysis has identified functional gene loss in *C. abortus* [9], and has shown that there is very little whole gene variation between S26/3 and LLG [6], in which the varying CDSs often represent differences in annotation rather than true coding differences, insertions or deletions. In order to assess any putative differences in functional gene content we compared the distribution of pseudogenes across the phylogeny. Starting with curated annotation of S26/3, LLG, AB7, and 1B representing each of the hierBAPS primary clusters, we identified a total of 51 pseudogenes within *C. abortus*. Sequence data from all other strains was then analysed to ascertain whether intact or disrupted versions of these genes were present (Fig. 3, Additional file 4).

From the sequences included in this study, 11 pseudogenes were found to be ancestral to the species including *pmp9* (*CAB273*) and *CAB529*, encoding the arginine repressor ArgR. One of the 11 pseudogenes (*CAB383A*, encoding a putative membrane protein) is found to have reverted to the predicted wild-type sequence in two strains due to variation within a homopolymeric tract. An additional five CDSs, including *CAB270* (*pmp8G*), have acquired disruptive mutations independently in both the major clade and cluster 3, resulting in their likely inactivation in all strains.

Of the predicted pseudogenes showing restricted phylogenetic distribution, the major clade has eight unique pseudogenes, predicted to encode hypothetical proteins or TMH/Inc family proteins. Cluster 3, comprising LLG and POS, contains nine unique pseudogenes, including two involved in biotin biosynthesis (*bioA*, *CAB688* and *bioD*, *CAB687*) and *CAB442*, a 4-alpha-glucanotransferase which is located adjacent to a type three secretion system operon. Cluster 2 has three unique pseudogenes, including that of *CAB551* (*guaB*) encoding inosine-5′-monophosphate dehydrogenase, meaning that the previously identified loss of purine nucleotide biosynthesis [9] is not a feature of the species, or even of the major clade. The variability in functionality within the *pmp* and TMH/Inc families has been previously noted [9]. Within the genomes analysed here, several *pmp* genes (*pmp8G* (*CAB270*), *pmp12G* (*CAB279*), *pmp13G* (*CAB281*) and *pmp15G* (*CAB283*)) and one TMH/Inc family protein-encoding gene (*CAB775*) are inactivated independently in various lineages. Variation in the length of homopolymeric tracts affecting *CAB279* (*pmp12G*), *CAB281* (*pmp13G*), *CAB383A* (encoding a putative membrane protein), *CAB596* (*pmp16G*), *CAB820* (encoding a conserved hypothetical protein) and *CAB852* (conserved membrane protein) are noted sporadically in several isolates, reiterating a possible role for phase variation and pseudogenisation in the ongoing evolution of *C. abortus* [9].

Searches for plasmid sequences within the *C. abortus* sequencing reads did not identify any putative plasmid related sequences, in contrast to other species within the *Chlamydiaceae*.

### Host-specific, geographical and temporal signals within *C. abortus* phylogeny

Discriminant Analysis of Principal Component (DAPC) was used to look for associations between population structure and animal source, geography and date of isolation. No significant clustering was identified for host or date. In the case of lineage and host, this may be expected since the sampling was biased towards ovines, and thus any signal may be obscured. However, although there are far fewer goat and cattle isolates compared to those from sheep, it is clear that they are nonetheless present throughout the phylogeny, and in PGs with isolate genomes from intensively farmed sheep.

Among the PGs identified, some geographical signatures can be identified (Fig. 1). DAPC analysis of the major clade revealed a statistically significant differentiation among strains from the different countries, above that identified with random continent assignment analysis. However, the signal is not as strong as could be expected from viewing the metadata, as strains from one country fall into more than one PG. Inspecting the phylogeny (Fig. 1), PG A is dominated by nine French isolates, but also contains three isolates from Tunisia and Namibia, representing Northern and Southern Africa. Several PGs (B, D and E) predominantly contain isolates from Germany, with two Greek isolates also within PG D. All the 17 UK isolates sequenced, as well as the UK reference strain S26/3, fall within PG F: these have been isolated over 30 years and are separated from each other by a maximum of 38 SNPs. Also within this PG are two German isolates collected in 1983 and 1999 and a US isolate from 1959. Isolates falling into PG G comprise international isolates from the USA (*n* = 3), Namibia (*n* = 2) and Germany (*n* = 1). PG G also shares a common ancestor with the UK dominated clade PG F.

From the current data, Germany (with 22 isolates) shows the highest diversity in *C. abortus* of the countries sampled (nucleotide diversity (Pi) = 4.17E-04; Additional file 5), with isolates within four PGs. UK strains show the lowest diversity (*n* = 18; Pi = 1.44E-05), with all the isolates falling in PG F. This analysis was supported by an AMOVA test that showed that as much variance can be explained by the diversity between isolates from different countries (47.2%) as can by the diversity within the same country (52.8%) (*p*-value = 0.001), supporting the observation that although tight clusters are found for some countries, such as the UK, other locations show higher *C. abortus* diversity.

For determination of temporal signal, the correlation between the root-to-tip distance and the year of isolation was calculated for the major clade, as well as for clusters 1 and 2 independently. A significant correlation was found only for cluster 2 (R^2^ = 0.47; *p*-value < 0.01), with isolate genomes separated by 0–82 SNPs, providing a most recent common ancestor (MRCA) of this clade estimated at 1722 CE (common era). Despite this correlation, we were unable to generate reliable estimates of temporal signal using BEAST.

## Discussion

This is the first whole genome-based international phylogenetic analysis of multiple isolates of *C. abortus*. The samples we sequenced are largely from affected farmed animals in Western and Mediterranean Europe, isolated between 1959 and 2011, and include all those which we could access. We also included a limited number of samples from Africa (Tunisia in the North and Namibia in the South) and the USA. The majority of the isolates we sequenced originated from sheep, which represent the major species of animal affected by this pathogen. The resulting disease is a consequence of the intensive management of these animals that occurs around lambing time, where animals are in close contact with one another and infection is easily spread between them. Goats are similarly affected, while the disease is much more sporadic in cattle, and as such we had access to only a few samples. One limitation of our study was that we were not able to obtain any isolates from other ruminant and non-ruminant species, such as deer and pigs, where the disease is only rarely reported and so our data refers largely to the population circulating within domesticated livestock.

Our data lead to several important observations. It is clear that the vast majority of *C. abortus* isolates circulating internationally show a low level of diversity, with only 724 SNPs separating the most distantly related isolates within the major lineage (clusters 1 and 2), and only 6,718 variable sites recorded within the whole phylogeny. This compares to 17,163 variable sites identified within the phylogeny of *C. trachomatis* (52 strains covering the species diversity) [11], and 47,710 variable sites identified within *C. psittaci* strains (13 diverse strains) [13]. Recombination was not found to have occurred between the isolates sequenced, although the low level of diversity may make recombination hard to detect.

Taking the phylogenetic tree as a whole, we can see that all sequenced isolates fall on two long branches separating LLG/POS (cluster 3) and the major lineage (clusters 1 and 2). This type of phylogenetic structure is a common feature of chlamydial phylogenies, indicative either of un-sampled diversity or, more likely, of evolutionary bottlenecks. In this case, bottlenecks appear to have occurred prior to the MRCA of the species, and also prior to expansion of the major clade. Such a scenario has also been put forward following a combined MLST/MLVA typing approach [19], and would concur with the lack of the virulence plasmid in all strains, within a species which has evidently evolved from an ancestor shared with the plasmid-carrying *C. psittaci* [18]. The structure of the major lineage is also interesting, being a polytomy indicative of a rapid expansion and diversification of closely related lineages over a similar time period.

While any dating within the phylogeny must be interpreted carefully, we have calculated the age of the MRCA of cluster 2 as being within the last 300 years, implying through extrapolation that the common ancestor of the major clade occurred several millennia ago. This data does not correlate with the previous estimate of the age of the whole species at 1881 CE, when it has been estimated to have split from its nearest species *C. psittaci* [18]. However, this published estimate was based on analysis of average mutation rates within the whole family *Chlamydiaceae*, and included only seven genomes from *C. abortus*. Sheep domestication occurred around 12,000 years ago in western Iran, Turkey, Syria and Iraq, from where they were brought 9,000 years ago into central Asia and then into Britain around 6,000 years ago, and this disease may have developed in parallel with domestication and the intensification of farming.

It is clear that the UK *C. abortus* population is extremely stable, with all UK isolates circulating in sampled herds over the last 30 years belonging to a single PG (F) with very low variation between isolates, indicative of clonal expansion. This is likely a reflection of the UK essentially being a closed flock as an island nation, with replacement animals being sourced at local markets. However, the foot and mouth disease (FMD) outbreak in 2001 revealed the extent of such animals movements, with animals being transported long distances all over Britain [41], which may explain the endemic nature of this disease throughout the UK as well as the clonality of the strain types observed. Two isolates obtained from a laboratory collection in Germany that were isolated from the placentas of sheep that had aborted as result of OEA, and one from the USA, were also found within the UK clade (PG F). Although the origin of these strains is unclear, from the genomic framework it is likely that these represent strain transfers from the UK abroad. Apart from these isolates, *C. abortus* from Germany occur in PGs B, D and E, with PGs B and D also displaying evidence of clonal expansion. Isolates from France are also largely clustered within PG A, with a subclade containing African strains having been transferred most likely through commercial trade. These French isolates demonstrate more diversity than the UK PG F, and single French isolates are also found within PGs B and C. Thus, while the movement of strains and, by implication, host animals, across the European mainland may be more common, national signatures often predominate, as also found in the recent MLST/MLVA study which documented the restriction of certain STs to specific geographical locations [19]. The influence of a possible unsampled pathogen reservoir within wild animals cannot be discounted. However, if this scenario were important then the diversity in this reservoir by implication must also be low, in any case suggesting that the extant *C. abortus* population has limited genomic flexibility. The diversity seen in cluster 3, representing Greek isolates LLG and POS, is also important to note, particularly as these are circulating in the same regions as isolates from the major clade [6, 19, 38, 42, 43]. Again, this hints at an historically more diverse population of *C. abortus* that has been through very severe population bottlenecks, revealing the deep divisions we now see in the species phylogeny.

Comparing the existing typing schemes for *C. abortus* to the whole genome SNP phylogeny shows that MLST and MLVA schemes broadly reflect the whole genome phylogeny. Although these schemes have limited discriminatory power, they can be useful in defining groups to a certain level, particularly in concert. While they do not sufficiently resolve the evolutionary history of the species, the lack of observed recombination within this species provides a more stable genomic background for such typing schemes. An alternative typing method should be possible through assaying a small number of variant sites, allowing discrimination of key European clades.

Of the 51 pseudogenes identified within the three clusters, 16 occurred ancestrally, nine are unique to the LLG/POS variant strains, and eight to the major clade. That most of these encode hypothetical proteins, and no phenotypic differences are described, means that the functions of these CDSs cannot be assessed. Disruption of CDSs encoding Pmp and Tmh/Inc proteins suggests that they are subject to specific selective pressure and their loss may be involved in niche adaptation. Biotin biosynthesis appears to have been lost in cluster 3 (LLG/POS), as it has also been lost from *C. caviae*, *C. trachomatis*, and *C. muridarum*, while being retained in *C. pneumoniae*, *C. pecorum* and the majority of *C. abortus*. Together with the lack of apparent recombination in this species it is clear that, apart from intrinsic variation in homopolymeric tracts, the possibilities of recovering mutations within the population through homologous recombination are limited. Since recombination appears to be a major evolutionary strategy in other species of *Chlamydia* this may suggest that *C. abortus* is approaching an evolutionary dead end, and may hint at opportunities to eradicate the disease.

## Conclusions

Our work provides the largest comparative genomic insights to date into the agent of an economically important agricultural disease. *C. abortus* possesses limited genomic diversity, within which evidence of recombination could not be found. The genome therefore appears to be very stable, and contains no plasmid. Several of the PGs identified have expanded regionally, indicating low levels of host transport within Europe, with very low levels of diversity particularly observed between strains from the UK.

## Additional files

Additional file 1: Isolates analysed in this study including source, sequence depth and MLVA and MLST sequence types. * coverage is low but even: SNPs can be confidently called in most cases. ** Different genome and year of isolation to our isolate of the same name. †Also sequenced by Joseph et al. 2015 but with lower coverage. †† MTs differ from those derived in Laroucau et al. 2009, and were derived from and checked using NGS data. (XLSX 19 kb)

Additional file 2: Recombination-associated genes identified within *C. abortus*. (XLSX 10 kb)

Additional file 3: Homoplasies identified within *C. abortus* isolates. It should be noted that some of the homoplasies derived for the *pmp* genes may have resulted from mis-mapping. *This is not within the quinolone resistance-determining region of *gyrA* [44, 45] and is not thought to confer any resistant phenotype. (XLSX 12 kb)

Additional file 4: Pseudogenes identified within *C. abortus* isolates. Cells highlighted in brown indicate pseudogenes. * Pseudogene annotations have been refined from those published in Sait et al. 2011, according to the latest genome draft version. ** LLG annotation shows that this is a longer protein, CAB617-618, also disrupted. (XLSX 13 kb)

Additional file 5: Nucleotide diversity between isolates by country. * This is 0.002218026 when including LLG/POS. (XLSX 9 kb)
